# OCOSO2: study protocol for a single-blinded, multicentre, randomised controlled trial assessing a central venous oxygen saturation-based goal-directed therapy to reduce postoperative complications in high-risk patients after elective major surgery

**DOI:** 10.1186/s13063-023-07689-z

**Published:** 2023-10-11

**Authors:** Bruno Pastene, Matthieu Bernat, Karine Baumstark, Karine Bezulier, Yann Gricourt, Jean-Manuel De Guibert, Aude Charvet, Manon Colin, Marc Leone, Laurent Zieleskiewicz

**Affiliations:** 1 Department of Anaesthesiology and Intensive Care Unit, Hôpital Nord, Hôpitaux Universitaires de Marseille, Marseille, France; 2 Centre for Cardiovascular and Nutrition Research (C2VN), INRA, Aix Marseille University, INSERM, Marseille, France; 3 Department of Epidemiology and Health Economy, Hôpitaux Universitaires de Marseille, Marseille, France; 4 Department of Anaesthesiology and Pain Management, Centre Hospitalo-Universitaire Carémeau, Nîmes and Montpellier University 1, Nîmes, France; 5 Department of Anaesthesiology and Intensive Care Unit, Institut Paoli Calmettes, Marseille, France

## Abstract

**Background:**

Fluid loading-based goal-directed therapy is a cornerstone of anaesthesia management in major surgery. Its widespread application has contributed to a significant improvement in perioperative morbidity and mortality. In theory, only hypovolemic patients should receive fluid therapy. However, to achieve such a diagnosis, a surrogate marker of cardiac output adequacy must be used. Current methods of fluid loading-based goal-directed therapy do not assess cardiac output adequacy. Nowadays, new devices make it possible to continuously monitor central venous oxygen saturation (ScvO_2_) and therefore, to assess the adequacy of perioperative cardiac output during surgery. In major surgery, ScvO_2_-based goal-directed therapy can be used to enhance fluid therapy and improve patient outcomes.

**Methods:**

We designed a prospective, randomised, single-blinded, multicentre controlled superiority study with a 1:1 allocation ratio. Patients to be included will be high-risk major surgery patients (> 50 years old, ASA score > 2, major intra-abdominal or intra-thoracic surgery > 90 min). Patients in the control group will undergo standard fluid loading-based goal-directed therapy, as recommended by the guidelines. Patients in the intervention group will have ScvO_2_-based goal-directed therapy and receive fluid loading only if fluid responsiveness and cardiac output inadequacy are present. The primary outcome will be the Comprehensive Complication Index on day five postoperatively.

**Discussion:**

This study is the first to address the issue of cardiac output adequacy in goal-directed therapy. Our hypothesis is that cardiac output optimisation during major surgery achieved by continuous monitoring of the ScvO_2_ to guide fluid therapy will result in a reduction of postoperative complications as compared with current goal-directed fluid therapy practices.

**Trial registration:**

ClinicalTrials.gov. NCT03828565. Registered on February 4, 2019.

**Supplementary Information:**

The online version contains supplementary material available at 10.1186/s13063-023-07689-z.

## Introduction

### Background and rationale

Fluid loading is a cornerstone of anaesthesia management in major surgery. Indeed, both fluid overload and hypovolaemia are deleterious for surgical patients [1, 2].

Several studies have assessed fluid loading based on clinical variables (arterial pressure, urine output, heart rate) and against goal-directed variables (cardiac output, stroke volume). These studies found a reduction in postoperative morbidity and length of hospital stay in goal-directed fluid therapy patients, including in long-term follow-up [3–6].

Since 2012, experts in the French Society of Anaesthesia and Intensive Care Medicine have recommended using goal-directed therapy for fluid loading in high-risk surgery patients [7]. These guidelines require the assessment of dynamic fluid responsiveness before administering fluid. If the patient is not a fluid responder, fluid loading is not administered. This strategy makes it possible to stop fluid loading as soon as it becomes deleterious.

However, it is not known whether fluid responsiveness alone is sufficient to determine the need for fluid loading. Indeed, with regard to the Frank-Starling law, fluid responsiveness is a physiological status, which means that there is no need to provide fluid in all responders [8]. Hypovolaemia is defined as a decrease in venous return, which is significant enough to cause an inadequacy of cardiac output. In theory, only hypovolemic patients should receive fluid loading. To achieve this endpoint, a surrogate marker of cardiac output adequacy should be used, such as serum lactate concentration or central venous oxygen saturation (ScvO_2_).

Serum lactate concentration is a late marker of cardiac output adequacy. In addition, it is not sufficiently accurate to detect slight changes in cardiac output. In contrast, ScvO_2_ is an excellent surrogate for detecting cardiac output variations. Today, new devices make it possible to monitor ScvO_2_ continuously and therefore to assess the adequacy of perioperative cardiac output. This facility could be used to enhance fluid therapy during major surgery and improve patients’ outcomes.

### Objectives

Our hypothesis is that using the continuous monitoring of ScvO_2_ to guide fluid therapy during major surgery will result in better cardiac output optimisation with a reduction in postoperative complications as compared with current goal-directed fluid therapy practices.

### Trial design

This study will be a prospective, randomised, single-blinded, multicentre controlled superiority study with a 1:1 allocation ratio.

## Methods

### Study setting

Three French hospitals in which major surgery is performed: Hôpital Nord (Assistance Publique Hôpitaux Universitaires de Marseille, Aix Marseille Université, Marseille France), Institut Paoli Calmette (Marseille, France), Hôpital Caremeau (Centre Hospitalier Universitaire de Nîmes, Nîmes, France).

### Eligibility criteria

Adult patients over 50 years of age with an American Society of Anesthesiologists (ASA) score of between 2 and 4 who are scheduled for elective major surgery. Elective major surgery is defined as abdominal or thoracic surgery with a duration of at least 90 min and requiring the use of invasive blood pressure monitoring and a central venous line.

### Interventions

#### Control group

Patients in the control group will be treated with standard care. Fluid loading will be performed in accordance with French guidelines [7]. The cardiac output and/or the stroke volume will be continuously monitored with FloTrac technology (pulse contour analysis, Edwards Lifesciences, USA) or CardioQ technology (oesophageal Doppler monitoring, Deltex Medical Group, UK).

**Fig. 1:**
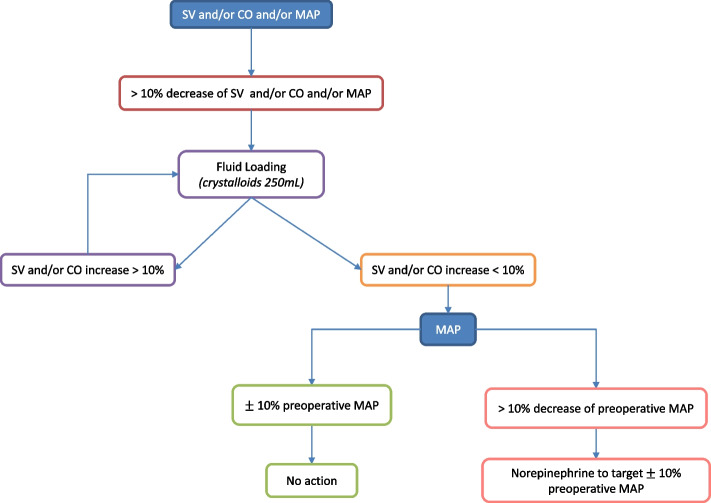
Overview of the control group protocol. Abbreviations: SV, stroke volume; CO, cardiac output; MAP, mean arterial pressure

If a diminution in cardiac output, stroke volume and/or mean arterial pressure of at least 10% of the initial values is observed, a rapid (5 min) fluid loading of 250 mL of balanced crystalloid solution will be administered. If the stroke volume and/or cardiac output increase by at least 10%, additional fluid loading will be administered. When the stroke volume and/or cardiac output do not increase or increase by less than 10%, fluid loading will be stopped. If the patient is not fluid responsive with a mean arterial pressure below 10% of the value of his or her preoperative mean arterial pressure, a diluted norepinephrine (16 µg mL^−1^) infusion will be started (Fig. 1).

#### Intervention group

Patients in the intervention group will be treated via an algorithm based on continuous perioperative ScvO_2_ monitoring (PreSep, Edwards Lifesciences, USA).

If ScvO_2_ is above 70%, cardiac output will be defined as adequate. A diluted norepinephrine infusion will be used to maintain a mean arterial pressure within a 10% variation of the value of the preoperative mean arterial pressure, regardless of fluid responsiveness status.

If ScvO_2_ is above 70%, but the need for norepinephrine is above 0.5 µg kg^−1^ min^−1^ to reach the blood pressure targets, a fluid loading of 250 mL of crystalloids will be administered. Administration will be repeated every 15 min until the norepinephrine dosage is below 0.5 µg kg^−1^ min^−1^.

**Fig. 2:**
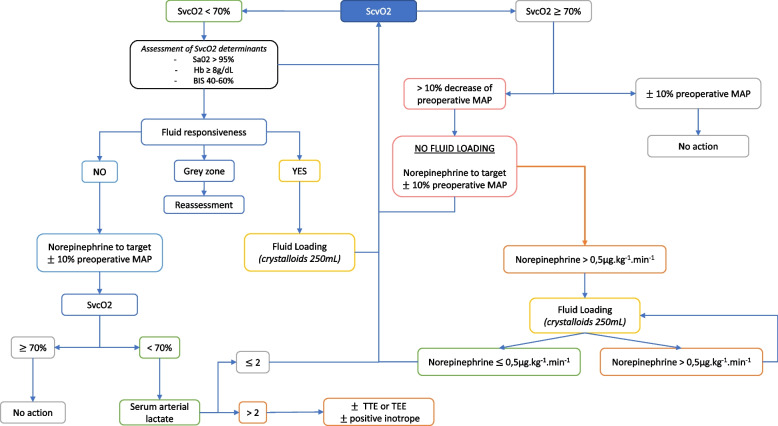
Overview of the intervention group protocol. Abbreviations: ScvO2, central venous oxygen saturation; SaO2, arterial oxygen saturation; Hb, haemoglobin; BIS, bispectral index; MAP, mean arterial pressure; TTE, transthoracic echography; TEE, transoesophageal echography

If ScvO_2_ is below 70%, cardiac output will be defined as inadequate. First, three out of the four determinants of ScvO_2_ will be assessed and corrected as needed (haemoglobin, arterial oxygen saturation and oxygen consumption through to the level of sedation). Then, fluid responsiveness will be assessed as described above. If the patient is fluid responsive, a fluid loading of 250 mL will be administered. If ScvO_2_ increases above 70%, fluid loading will be stopped. If not, the fluid loading will be administered as long as ScvO_2_ remains below 70%, with positive fluid responsiveness. If the patient is in the “grey zone” (when the fluid responsiveness assessment is not informative), the patient will be reassessed after 5 min (Fig. 2).

### Outcomes

The primary outcome will be the Comprehensive Complication Index (CCI) on day five after surgery [9]. CCI is a validated score for postoperative morbidity, ranging from 0 (no complication) to 100 (death). This score is based on the Clavien-Dindo classification of postoperative complications, and it weighs the severity of the complications by the resources needed to treat them [10]. CCI offers a sensitive endpoint and is particularly suitable for randomised controlled trials [11].

Regarding secondary outcomes, our hypothesis is that our experimental strategy will reduce the quantity of fluid administered during surgery. Therefore, the secondary outcomes will be the total amount of fluid administered perioperatively and the lung ultrasound score (LUS). LUS is a simple, fast and reproducible measure to assess the potential harm of excessive fluid administration [12]. To assess long-term outcomes, the CCI will be measured at days 15 and 30.

### Participant timeline

Patients will be recruited during their pre-anaesthesia consultation. The protocol will be explained, and written consent obtained. On the day of surgery, the patients will be randomly allocated to either the control group or the experimental group. During surgery, haemodynamic, ventilatory and pharmacologic data will be collected. In the post-anaesthesia care unit (PACU), an arterial and a venous blood gas analysis and an LUS will be performed in patients after tracheal extubation.

The patients will be monitored daily during the 5 days following surgery. A phone call will be made on day 15 and on day 30 to collect information about potential postoperative complications. The duration of participation for each patient included in the study will be 30 days. Patients cannot be included in another study for 30 days after their randomisation.

### Sample size

A sample size calculation was made from published data [9, 11]. In order to show a 10% difference in CCI between groups on day five after surgery, with a 0.9 power and a 0.05 alpha risk, 100 patients were needed per group. Considering a potential 10% loss to follow-up, we included 220 patients (110 patients per group). We chose a 10% difference in CCI because this represents a clinically significant decrease in postoperative complications (one grade on the Clavien-Dindo classification).

### Recruitment

Patients will be recruited during the anaesthesia consultation by a senior anaesthesiologist once the protocol has been explained and their written consent obtained.

### Allocation

Randomisation will be performed with the block randomisation technique to reduce bias and achieve balance in patient allocation. Randomisation will be stratified by the treatment centre to reduce selection bias. Randomisation will be performed on surgery day by the attending anaesthesiologist with the nQuery Advisor software (v.7.0, Statsols, USA).

### Blinding

Due to the study design, the attending anaesthesiologist can’t be blinded to the study intervention. Therefore, double blinding will not be feasible. However, the patient is blinded to his study group and postoperative data collection and statistical analysis will be performed by investigators blinded to the group allocation of each patient. Since the attending physicians is unblinded to the study intervention, no emergency unblinding strategy has been developed.

### Data collection, management and analysis

All data will be collected in real-time by the clinical research team of the participating centres using a case report form. The variables to be collected are as follows:

- Demographics (age, gender, ASA score, height, weight, BMI).
- Comorbidities: Charlson comorbidities score and preoperative score to predict postoperative mortality (POSPOM).
- Surgical risk (ACS NSQIP surgical risk evaluation) and surgical procedure.
- Perioperative:
  - ◦ Haemodynamic (continuous): mean, systolic and diastolic blood pressure, heart rate, cardiac output, stroke volume, stroke volume variation and ScvO_2_ for the patients in the experimental group.
  - ◦ Respiratory (every 30 min): tidal volume, respiratory rate, FiO_2_, positive end-expiratory pressure (PEEP).
  - ◦ Neurologic (every 30 min): bispectral index (BIS), suppression ration (SR).
- At the end of surgery: total volume of fluids administered (crystalloids and blood products), type and total amount of anaesthesia drugs used, use of an epidural analgesic.
- In the PACU: arterial and venous blood gas analysis, LUS.
- Postoperative (daily): type and severity of postoperative complications until day 5 postoperative. CCI score calculation on day 5.
- At day 15 postoperative: collection of postoperative complications between day 5 and day 15 and CCI score calculation on day 15.
- At day 30 postoperative: collection of postoperative complications between day 15 and day 30 and CCI score calculation on day 30.

Data entry will be performed using REDCap software. Data analysis will be performed by a statistician from the clinical research team, using SPSS software (v 17.0, IBM, USA).

Table 1 shows the schedule of enrolment, intervention and assessment during the study period.

**Table 1:** Schedule of enrolment, interventions and assessments

	**Study period**							
	**Enrolment**	**Allocation**	**Post-allocation**					**Close-out**
**Timepoint**	**Pre-anaesthesia consultation**	**Surgery day**	***t***_***1***_ *During surgery*	***t***_***2***_ *PACU*	***t***_***3***_ *D1 to D5 postop*	***t***_***4***_ *D15 postop*	***t***_***4***_ *D30 postop*	**D30 postop**
**Enrolment:**								
Eligibility screen	X							
Informed consent	X							
Allocation		X						
**Interventions:**								
Control group: standard care			X					
Intervention group: ScvO_2_-guided GDT			X					
**Assessments:**								
Demographics, comorbidities, surgical risk		X						
Haemodynamic, respiratory and neurologic variables Total fluid administration			X					
Arterial and venous blood gas analysis, LUS				X				
Postoperative complications, CCI					X	X	X	
Close-out form								X

Abbreviations: *PACU* post-anaesthesia care unit, *D* day, *GDT* goal-directed therapy, *LUS* lung ultrasound score, *CCI* Comprehensive Complication Index

### Statistical methods

Statistical analysis will start only after the database has been validated (all queries answered, database coherence controlled). The statistical analysis will be blinded (no explicit identification of groups). The methodology and statistical analysis will be performed in accordance with the CONSORT statement [13]. The population analysis will be performed according to the intention-to-treat (ITT) principle. A secondary, per-protocol population analysis will be performed if needed. If present, missing data will be handled via multiple imputation by chained equations. Categorical variables will be reported as count (%) and continuous variables as mean (± standard deviation (SD)) or median (25th–75th quartile range). The presence of a normal distribution will be verified using the Kolmogorov-Smirnoff test. We will use the *t*-test to assess differences between parametric continuous variables, the Mann–Whitney test for non-parametric variables, the *χ*^2^ test for categorical variables and the Fisher exact test for 2 × 2 tables. No correction for multiple testing will be done. A two-sided *p* < 0.05 will be considered statistically significant. Stopping of the trial could also be based on interim data analysis if clearly one treatment is better than the other, but no interim analysis is planned.

### Monitoring

In accordance with the Helsinki Declaration and French law, the research team will declare adverse events without delay to the study sponsor (Assistance Publique Hôpitaux de Marseille). Participation in the study will be clearly stated in the patients’ medical files. The data monitoring and the coordinating committees from the study sponsor will perform annual data-monitoring sessions for quality control and trial conduct auditions during the study period.

## Ethics and dissemination

### Research ethics approval

This study was approved by an institutional review board (Comité de Protection des Personnes SUD-EST VI) on December 9, 2019. All participants will be able to consent to the study and written consent will be obtained from all participants. Minors and patients unable to understand the study or under guardianship will not be included.

### Protocol amendments

Two amendments have been made to the original protocol. The first amendment removed the diagnosis of heart arrhythmia from the exclusion criteria, because stroke volume variation was not used in the study to assess fluid responsiveness. Due to safety concerns, the second amendment added the 250 mL fluid loading in the experimental group should the norepinephrine dose exceed more than 0.5 µg kg^−1^ min^−1^. All participating centres were notified without delay by the study sponsor of any protocol amendments. If a major deviation from the study protocol should happen, it will be documented by the investigators with a Breach Report Form.

### Consent or assent

All participants will have consented to the study. Minors and patients unable to understand the study or under guardianship will not be included.

### Confidentiality

The database will be anonymous and encrypted to ensure data safety and patient confidentiality.

### Declaration of interests

Professor Leone reports personal fees received from Aspen, AMOMED, LFB, Ambu and Gilead outside of the submitted work.

Professor Zieleskiewicz reports personal fees received from General Electric Healthcare outside of the submitted work.

Dr Pastene reports personal fees from Edwards Lifesciences outside of the submitted work.

Dr Bernat, Dr Baumstark, Dr Bezulier, Dr Gricourt, Dr De Guibert, Dr Charvet and Dr Colin have nothing to disclose.

### Access to data

The datasets analysed during the current study and statistical code will be available from the corresponding author, BP, on reasonable request, as is the full protocol.

### Ancillary and post-trial care

Patients enrolled in the study will be covered by indemnity for negligent harm through the standard indemnity arrangements of French national health insurance. Hôpitaux Universitaires de Marseille has insurance to cover non-negligent harm associated with the protocol.

### Dissemination policy

The authors aim to publish the present protocol in a high-impact, peer-reviewed anaesthesiology journal. The results will be published even in the event of non-significant results.

### Supplementary Information

**Additional file 1.**

## Data Availability

The datasets analysed during the current study and statistical code will be available from the corresponding author, BP, on reasonable request, as is the full protocol.
